# Editorial: Regulation of Light-Harvesting Systems During Acclimation of Photosynthetic Organisms

**DOI:** 10.3389/fpls.2022.914047

**Published:** 2022-05-05

**Authors:** Thomas Roach, Eunchul Kim, Lijin Tian, Bernard Lepetit

**Affiliations:** ^1^Department of Botany, University of Innsbruck, Innsbruck, Austria; ^2^Division of Environmental Photobiology, National Institute for Basic Biology, Okazaki, Japan; ^3^Key Laboratory of Photobiology, Institute of Botany, Chinese Academy of Sciences, Beijing, China; ^4^Department of Plant Ecophysiology, University of Konstanz, Konstanz, Germany

**Keywords:** non-photochemical quenching (NPQ), chlorophyll, acclimation (photosynthesis), light-harvesting, stress

Light-harvesting by photosynthetic plants, algae, and bacteria is the primary process that powers almost all of earth's ecosystems, as well as producing O_2_ and removing CO_2_ from the atmosphere. Since light conditions are dynamically fluctuating, both in terms of intensity and wavelength, light-harvesting systems are dynamically regulated by various mechanisms across short and long time scales for achieving efficiency, and preventing excess energy absorption from producing high amounts of reactive oxygen species (ROS), leading to photooxidative stress. Short-term acclimation includes excess energy dissipation (energy-dependent quenching, qE) and energy redistribution of the light harvesting apparatus (state transitions, qT), which can be activated in response to the pH of the lumen and electron chain redox state, respectively, thus forming direct feedback to energetic statuses. Such processes occur amongst the constant fluctuation of transcription, translation and post-translational modifications that tune the light harvesting apparatus to current conditions.

Excess energy dissipation involves excitation quenching induced by the interactions between chlorophylls and carotenoids, such as the lutein located in the light-harvesting complex, but exact physical mechanisms are unclear. The article by Gray et al., “*Trivial Excitation Energy Transfer to Carotenoids is an Unlikely Mechanism for Non-Photochemical Quenching in LHCII*” provides evidence that trivial geometric modulation and Coulomb-mediated energy transfer to an optically forbidden lutein S1 state are insufficient to activate the energy dissipation in the LHCII, in contrast to what has been previously postulated. The authors therefore suggest that the energy dissipation mechanism likely involves short-range intermolecular interactions, which are more sensitive to geometric modulation.

The role of the violaxanthin xanthophyll cycle in plants and the diadinoxanthin cycle in diatoms in NPQ is well-established. In the article “*The Loroxanthin Cycle: A New Type of Xanthophyll Cycle in Green Algae (Chlorophyta)*,” van der Berg and Croce demonstrate that in the unicellular model green alga *Chlamydomonas reinhardtii*, the hydroxylated form of lutein, loroxanthin, accumulates under low light conditions, including the night. The authors therefore suggest that as part of acclimation a lutein-loroxanthin cycle exists in this alga, alongside the ubiquitous violaxanthin-zeaxanthin cycle.

How various light-harvesting systems are regulated, which is critical for maintaining photosynthetic efficiency under naturally dynamic light conditions, is not yet fully understood due to diverse light-harvesting systems and their complexity. In “*Structural Diversity of Photosystem I and Its Light-Harvesting System in Eukaryotic Algae and Plants*” Bai et al., review the structural details of the PSI tetramer in cyanobacteria and the PSI-LHCI and PSI-LHCI-LHCII supercomplexes from different algae and plants, and then discuss the diversity of PSI-LHCI in oxygenic photosynthesis organisms, thus providing an up-to-date overview of this light-harvesting system.

In addition, regulation of light-harvesting is largely interconnected with acclimation to other abiotic constraints, such as temperature changes and nutrient availability. In oceans, iron availability is often limiting to algal growth, requiring much of the absorbed light energy to be dissipated as heat (i.e., qE) rather than being useful for driving photosynthesis. Buck et al. investigate the importance of qE *via* Lhcx proteins under low iron availability in diatoms in “*Impact of Lhcx2 on Acclimation to Low Iron Conditions in the Diatom Phaeodactylum tricornutum*.” They demonstrate that a typical photosynthetic low iron acclimation syndrome is independent of the parallel up-regulation of qE capacity at PSII. However, qE lowers ROS accumulation at PSII under iron limitation, while PSI generated ROS production is independent of qE.

Due to the strong light absorber chlorophyll, plants are very efficient in absorbing sunlight and, as mentioned, release a surplus of absorbed energy, not used in photosynthesis, as heat. Crops with less chlorophyll absorb less light, thus have increased albedo and could be “climate-friendly.” In the article “*Does Fluctuating Light Affect Crop Yield? A Focus on the Dynamic Photosynthesis of Two Soybean Varieties*,” Salvatori et al., show that a chlorophyll deficient soybean mutant (MinnGold) is less efficient, relative to wild type, under light fluctuations of high intensity, but not when the light intensity of fluctuation is lower. This reveals the complexity of light harvesting, and illustrates that reducing chlorophyll content may have surprising consequences, such as not leading to tolerance of higher light intensity.

Artificially tuning photosynthesis may help tackle social challenges, such as energy and food crisis, and global warming. However, if we want to produce higher-yielding crops, high-CO_2_-fixing plants or algae, and better predict the photosynthetic productivity in various ecosystems, we need to continue researching and improving understanding of light harvesting regulation.

## Author Contributions

All authors listed have made a substantial, direct, and intellectual contribution to the work and approved it for publication.

## Conflict of Interest

The authors declare that the research was conducted in the absence of any commercial or financial relationships that could be construed as a potential conflict of interest.

## Publisher's Note

All claims expressed in this article are solely those of the authors and do not necessarily represent those of their affiliated organizations, or those of the publisher, the editors and the reviewers. Any product that may be evaluated in this article, or claim that may be made by its manufacturer, is not guaranteed or endorsed by the publisher.

